# Intelligent Evacuation Sign Control Mechanism in IoT-Enabled Multi-Floor Multi-Exit Buildings

**DOI:** 10.3390/s24041115

**Published:** 2024-02-08

**Authors:** Hong-Hsu Yen, Cheng-Han Lin

**Affiliations:** 1 Department of Information Management, Shih Hsin University, Taipei 116, Taiwan; 2 Department of Computer Science and Engineering, National Chung Hsing University, Taichung 402, Taiwan; herryeric88@gmail.com

**Keywords:** evacuation sign, multi-floor multi-exit building, congestion-aware evacuation, smoke-safe evacuation, optimization

## Abstract

In contemporary evacuation systems, the evacuation sign typically points fixedly towards the nearest emergency exit, providing guidance to evacuees. However, this static approach may not effectively respond to the dynamic nature of a rapidly evolving fire situation, in particular if the closest emergency exit is compromised by fire. This paper introduces an intelligent evacuation sign control mechanism that leverages smoke and temperature sensors to dynamically adjust the direction of evacuation signs, ensuring evacuees are guided to the quickest and safest emergency exit. The proposed mechanism is outlined through a rigorous mathematical formulation, and an ESP heuristic is devised to determine temperature-safe, smoke-safe, and congestion-aware evacuation paths for each sign. This algorithm then adjusts the direction light on the evacuation sign to align with the identified evacuation path. To validate the effectiveness of this approach, fire simulations using FDS software 6.7.1 were conducted in the Taipei 101 shopping mall. Temperature and smoke data from sensor nodes were utilized by the ESP algorithm, demonstrating superior performance compared to that of the existing FEL algorithm. Specifically, the ESP algorithm exhibited a notable increase in the probability of evacuation success, surpassing the FEL algorithm by up to 34% in methane fire scenarios and 14% in PVC fire scenarios. The significance of this improvement is more pronounced in densely congested evacuation scenarios.

## 1. Introduction

In recent years, with the rise of the Internet of Things (IoT) and the accelerating commercialization of related IoT products, IoT-assisted fire evacuation can evacuate more occupants than traditional evacuation signage systems. The loss of life and property in buildings due to fires is significant. According to statistics from the National Fire Protection Association (NFPA) in 2022, there were 1,504,500 fire incidents in the U.S.A., with 3790 civilian deaths [1]. Current fire prevention measures in buildings primarily rely on smoke detectors and automatic sprinklers, with fixed signs indicating the direction to a specific evacuation exit. According to Taiwan’s current building fire regulations, high-rise buildings are required to have at least two evacuation routes, and large shopping malls have more than two escape doors. For example, Taipei 101’s shopping mall has four evacuation exits. However, fixed evacuation signs may mistakenly guide people towards exits affected by spreading fires, leading to a greater number of casualties. Therefore, developing evacuation routes tailored to different fire scenarios is crucial for successfully evacuating the public.

According to a fire research report [2], a fire can spread rapidly within a few minutes. The temperature can reach 500 °F within 3 to 4 min and a more dangerous flashover temperature of 1100 °F within five minutes. The human body can only withstand temperatures up to 212 °F or 100 °C [2]. Additionally, another study [3] found that exposure to a fire with a temperature of 350 °F for three minutes can lead to death due to thermal injuries. Research [4] indicates that the critical rescue time in a fire is 5.5 min; beyond this time, the fire may spread to another building.

In addition to high temperatures, another more deadly hazard in fires is thick smoke. According to a research report from the United States Fire Administration (USFA) [5], looking at the casualty rates from 2017 to 2019, injuries caused by respiratory tract irritation and poisoning due to thick smoke accounted for 50% of the total, while burns caused by high temperatures accounted for 30%. The main reason is that the speed of smoke spread is much higher than the speed of heat convection. Moreover, thick smoke not only causes difficulty in breathing but also impairs visibility, leading to dizziness and loss of orientation, and increases one’s risk of getting trapped in the fire. Additionally, fire smoke often contains toxic gases (e.g., carbon monoxide, CO). CO can quickly induce unconsciousness in evacuees. For example, if the concentration of carbon monoxide reaches 0.1%, evacuees can lose consciousness within one to two minutes [6]. These factors significantly reduce the effective evacuation time and probability of survival for those trapped in a fire. The above fire research data indicate that the evacuation time for individuals trapped in a fire is extremely limited. The spread of a fire can lead to drastic changes in temperature and smoke concentration along evacuation routes in a very short time.

Fire detection and evacuation have been an important current issue in multi-floor building construction. In modern multi-floor buildings, temperature and smoke sensors are deployed to detect the fire. When a sensor detects that the temperature or smoke is above a threshold, a fire alarm is triggered, and occupants of the building evacuate by following the evacuation sign on the wall to the nearest emergency exit. However, the evacuation direction on the evacuation sign is fixed. Occupants following the evacuation sign to the nearest emergency might be stuck on the route when the fire spreads to the emergency route. This calls for an intelligent evacuation path that can bypass the fire zone to the emergency exit in a timely manner. In other words, the planning of evacuation routes must be based on the real-time changes in the fire for people to avoid dangerous areas and quickly reach evacuation exits. This evacuation principle becomes even more challenging in high-rise buildings or large complex malls that require more evacuation time. Compared to evacuating a single floor, evacuating from higher floors involves three-dimensional spatial route planning, including the use of stairs for both ascending and descending.

**Figure 1:**
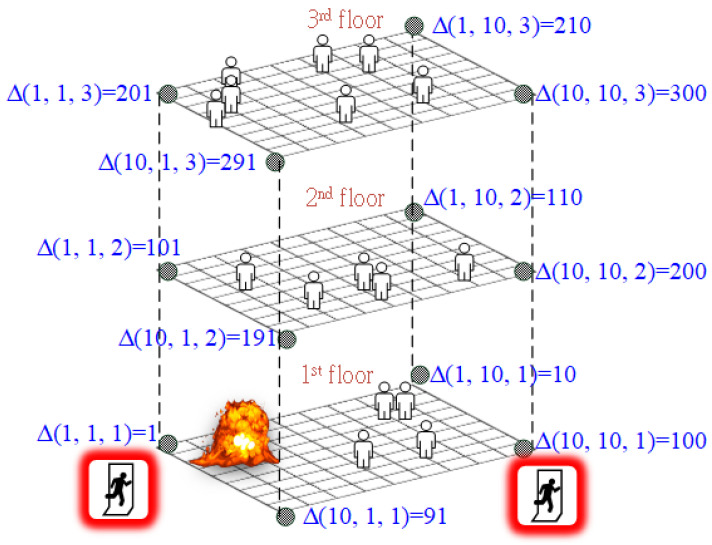
Fire evacuation route planning in multi-story multi-exit building.

Figure 1 illustrates an example of a three-story building without compartments. In this example, each floor is divided into a 10 × 10 grid, and each intersection represents the location where sensors are placed. In this three-story example, a total of three hundred sensors are placed to monitor temperature and smoke values at their respective locations. Here, Δ(*I*, *j*, *k*) represents the node ID of the sensor node at coordinates (*i*, *j*) on floor k of the building. The node IDs are assigned starting from the upper-left corner of the first floor, moving to the bottom right, and then progressing to the second and third floors. Additionally, there are four staircases connecting the upper and lower floors of the building. For example, the connection between node ID 210 and node ID 110 links a staircase from the third floor to the second floor, while the connection between node ID 110 and node ID 10 links a staircase from the second floor to the first floor. Therefore, in the example of Figure 1, people on the third floor have access to four staircases to evacuate down to the next floor.

In Figure 1, the fire starts near the evacuation exit at node ID 1 on the first floor. It can be anticipated that as the fire spreads, the evacuation exit at node ID 1 will soon be filled with smoke and become impassable. Therefore, the key to successful evacuation lies in how to quickly people are guided inside the building to the evacuation exit at node ID 100. Several steps are crucial to have real-time awareness of the fire’s spread in Figure 1. First, the on-site installation of temperature and smoke sensors is necessary. Given the rapid spread of the fire, temperature and smoke sensors provide real-time information at regular intervals (e.g., every minute). Second, all sensors must continuously collect temperature and smoke data, and real-time evacuation route calculations must be performed based on this data. This information is then used for real-time evacuation route calculations, and the results are communicated to the evacuees. Third, the calculated evacuation routes need to be promptly communicated to the trapped occupants. Note that these three stages must be executed repeatedly in a short timeframe to identify immediate evacuation routes in response to the latest changes in the scene of the fire.

How to communicate the calculated evacuation routes to evacuees in a timely manner is a challenging problem. According to fire safety rules, evacuation signs must be deployed in buildings to direct occupants to emergency exits when there is a fire alarm. Existing evacuation signs on walls are fixed and always point toward the nearest emergency exit in buildings. When there is a fire alarm, an evacuee will follow the evacuation sign to the nearest exit. However, following the evacuation sign to the nearest exit might lead to a fire zone. This results in more injuries and death while evacuating. A better evacuation sign would be able to guide evacuees to a safe evacuation route based on the fire’s current progression. In other words, instead of a traditional evacuation sign that shows a fixed evacuation direction, dynamic evacuation signage that adapts to the fire’s progression and dynamically points to a safe emergency route should be adopted.

Besides smoke and heat hazards, evacuation congestion is another deadly threat. In the example in Figure 1, the evacuation exit at node ID 100 is the only safe exit after the fire spreads to the evacuation exit at node ID 1. It can be anticipated that the staircase from node ID 200 on the second floor to node ID 100 on the first floor will be very congested because the evacuees on the second floor and third floor will likely choose this staircase to evacuate to reach the evacuation exit at node ID 100. The congestion at this staircase will not only slow down their evacuation speed but can also lead to a stampede. The physical damage caused by stampedes prevents people from evacuating and causes them to become injured or suffocate [7]. One possible solution to this is to guide some of the evacuees to evacuate using other staircases (e.g., the staircases from node ID 110 to node ID 10). Hence, a safe evacuation system should consider the threats from heat, smoke, and congestion simultaneously.

**Figure 2:**
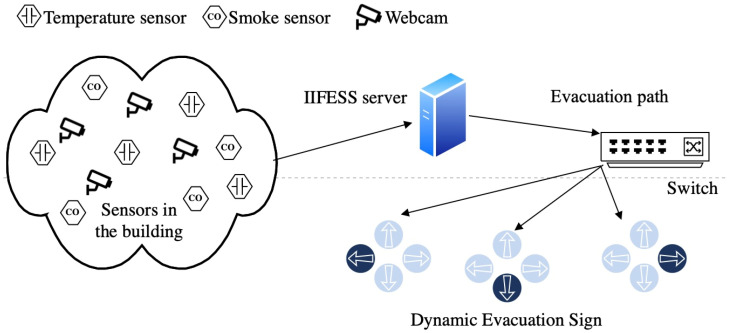
Intelligent IoT-enabled Fire Evacuation Signage System (IIFESS).

To tackle these three threats at the same time, we propose the architecture of the Intelligent IoT-enabled Fire Evacuation Signage System (IIFESS) as shown in Figure 2. In the IIFESS architecture, each floor of the building is equipped with smoke and temperature sensors. These IoT-enabled temperature and smoke sensors regularly (e.g., every minute) transmit sensed data to the IIFESS server through wired or wireless signals. Once the IIFESS server collects the sensed data at regular intervals, it utilizes the algorithm proposed in this paper to calculate real-time evacuation directions for each intelligent evacuation sign. In addition, without prior knowledge of the evacuees’ location, we propose a congestion-aware evacuation signage control scheme by using the evacuation path information from each evacuation sign to circumvent the congestion that occurs when evacuating. The IIFESS developed in this paper can provide real-time evacuation routes for groups (intelligent evacuation signs).

In this paper, we first propose the mathematical models to capture this evacuation signage control problem in multi-floor multi-exit buildings and then develop a temperature-aware, smoke-aware, and congestion-aware algorithm for safe evacuation.

We summarize the contributions of this paper as follows.

A. Dynamic Evacuation Signage: Existing evacuation signs on walls are fixed and always point toward the nearest emergency exit in buildings. However, following an evacuation sign to the nearest exit might lead one to a fire zone. In this paper, we propose dynamic evacuation signage that can adapt to the fire’s progression and dynamically point to a safe emergency route.
B. IoT-enabled Evacuation Sign Control Mechanism: In the IoT-enabled evacuation approaches in [8,9], the evacuation route is sent to the evacuees’ phones. In other words, the evacuation route can only be sent to occupants who have installed the evacuation app. However, in most cases, occupants will not have installed the app and the evacuation signs on the wall will be the only way to guide them in an evacuation. In this paper, we propose an intelligent evacuation sign control mechanism to set the direction of evacuation signs to adapt to a fire’s progression in an evacuation.
C. Consideration of the Smoke and Burn Hazards in Fires: The spread of a fire can lead to drastic changes in temperature and smoke concentration along evacuation routes in minutes; burn injuries and smoke injuries are the two most common deadly factors during a fire evacuation. In this paper, temperature and smoke hazards are considered when dynamically setting the direction of the evacuation signs for safely and quickly evacuating the building.
D. Consideration of the Congestion Hazard in Fires: In an evacuation, congestion will lead to stampedes, which will incur crushing injuries or death. In this paper, the congestion hazard is considered, and a congestion danger index is proposed in our evacuation mechanism to guide evacuees away from congested sites. In addition, a recursive mathematical formula is proposed to calculate the evacuation time with respect to the number of evacuees, which captures the congestion hazard during an evacuation.

## 2. Related Works

With the rapid development of sensors and the IoT, real-time temperature and smoke data are available to enable dynamic evacuations in state-of-the-art IoT buildings. Numerous prototypes of Internet of Things (IoT)-enabled fire detection systems have been developed. For instance, in [10], an Arduino board was designed to incorporate temperature sensors, flame sensors, and a ZigBee module to facilitate home-based fire detection and alert systems. Similar systems utilizing wireless sensor networks and GSM communication modules for home-based fire detection are proposed in [11]. In another IoT prototype by [12], the toxic gas concentration is monitored, and a ventilation system is activated when the toxic gas levels in an industrial environment become elevated.

Compared to a fixed signage system, a dynamic signage system that can provide time-variant evacuation guidance according to the dynamic evolution of an incident has a better evacuation performance with a decreased evacuation time and decreased number of fatalities [13]. A fire evacuation signage framework is proposed in [14] for the real-time control of evacuation signs during a fire’s progression. Several microcontroller-based evacuation signage prototypes based on Arduino are proposed in [15,16]. In these signage prototypes, the ZigBee sensor sends a fire alarm signal to the server when the collected data are above a threshold.

How the color, size, position, and shape of emergency exit signs affect people’s understanding of and compliance with the direction indicated by evacuation signs are hot topics in existing research. It has been verified that the signage installation position and the arrow type on signs are important factors in determining evacuees’ understanding of and compliance with evacuation directions [17]. Evacuation signs that are placed high up have a positive effect on people’s evacuation route choices [18]. Ref. [19] similarly reports that evacuation signs on walls are more efficient than evacuation signs on the ground in terms of evacuation time. Ref. [20] reports that evacuation signs on walls are more likely to be perceived by evacuees than hanging evacuation signs.

It has been reported that an evacuation sign with a green arrow is the best for catching one’s eye [21]. Similar results are also confirmed in [22] that green evacuation signs are better than red evacuation signs in group evacuations. An interesting result was found in [23], which showed that most evacuees follow green evacuation signs even though they think evacuation signs should be red. They found out that evacuees move faster when the size of exit signs was increased than when the brightness was increased [24]. More recently, a three-dimensional evacuation sign structure has been proposed in [25], and they show that evacuees’ response time to a three-dimensional evacuation sign is significantly faster than that to a two-dimensional evacuation sign. Negative or dissuasive signage is studied in [26]. They found out that red flashing lights alert occupants to specific emergency signs and a red X placed across an exit sign clearly conveys one should not use that emergency exit.

The differences between individuals and groups in detecting and accepting the information conveyed by a signage system has been studied in [18]. They found out that the signage detection and acceptance probability for an individual is larger than that for a group because of the social aspect of groups. In [27], they study the effect of disturbers on evacuating signage guidance. They show that disturbers who always move in the opposite direction of evacuation signs have a strong impact on evacuees who do not see the evacuation signs. However, when evacuees see an evacuation sign, the evacuation signage determines their direction. This suggests more signs should be added in various positions to prevent evacuation signs being covered up by a crowd. The relationship between smoke density and evacuation speed has been studied in [28]. They found out that evacuation speeds according to the smoke density vary depending on the presence/absence of illumination and evacuation signs.

In [29], a BIM-controlled signage system is proposed to activate/deactivate exit signs to help in an evacuation. This system uses the returned signal from the fire sensor to detect the fire zone, and then the shortest path algorithm that avoids the fire zone is selected to activate/deactivate the exit signs that lead to the shortest route. A similar study controlling evacuation signs for a fire evacuation in an underwater tunnel is proposed in [30]. Virtual reality (VR) fire evacuation experiments are performed in [31] and show that adaptive emergency signs can be used to evacuate people more efficiently than traditional fixed emergency signs. Then, evacuation routes are calculated to avoid fire zones to prevent thermal damage. In these studies, fire sensors only report the presence or absence of a fire alarm. The temperature and smoke data from fire sensors are not used to compute the different levels of fire hazards on evacuation routes. An interesting fire experiment was conducted at Ikea in Sweden [32]. It showed that most of the evacuees ignored signs and ran out of the building, passing a small fire on the evacuation route. Additionally, a fast evacuation route that is within one’s tolerance for temperature and smoke might be classified as unsafe, slowing the evacuees’ evacuation speed. Hence, for a fire progressing quickly, fire hazards and evacuation speed need to be considered simultaneously to safely evacuate as many evacuees as possible.

To verify the evacuation success rate in dynamic evacuation signage systems, fire simulations are used to replicate the spread of smoke and heat during a fire incident. The Fire Dynamics Simulator [33], developed by the United States Department of Commerce’s NIST (National Institute of Standards and Technology), is a widely recognized tool in fire engineering for simulating the transport of smoke and heat in fires. In a study conducted by [8], it was confirmed that the smoke danger zone in a large shopping mall is more extensive than the heat danger zone, as demonstrated using FDS simulations.

Most fire-related fatalities result from inhaling toxic gases and oxygen depletion. In a study by [34], real fire experiments were conducted within an actual healthcare facility equipped with a sprinkler system. The outcomes revealed that the sprinkler system effectively suppressed the fire, reducing the risk of burns. However, it could not eliminate the risk of incapacitation due to toxic gases. Assessing the toxicity of fire-generated gases is crucial for planning safe evacuation routes. In research conducted by [35], it was highlighted that the two most harmful toxic gases for occupants are carbon monoxide (CO) and hydrogen cyanide (HCN), both of which can lead to rapid incapacitation and even death. These toxic gases can be categorized into two types: asphyxiants and irritants. Ref. [36] identified common asphyxiant gases and irritants in fire gases, while the International Standards Organization (ISO) document 13571 [37] introduced the Fractional Effective Dose (FED) index as a standard for quantifying the toxicity of fire-generated gases.

Most evacuation route algorithms are based on the shortest path algorithm. In studies by [38,39,40], a shortest-path-based algorithm that considers risk factors such as temperature, visibility, and CO density is used to guide evacuees to safe exits. Dijkstra’s shortest path algorithm is used to identify the best route based on the risk score associated with each link. In [41]’s research, a comparison between the safest and shortest paths revealed that the safest route is often longer than the shortest route in most tested scenarios. In [15], Dijkstra’s shortest path algorithm is performed on a server to determine the evacuation route, and a control signal is sent to an 8 × 8 RGB Arduino matrix panel to show the evacuation direction. An individual evacuation algorithm was proposed for multi-exit, multi-floor buildings based on real-time temperature data [9] and smoke data [8] from sensors.

Besides heat and smoke hazards, crowding is another threat that leads to injuries and death in fire evacuations. People-counting technology can help to sense overcrowded areas and evacuation algorithms can leverage this to avoid overcrowded areas when creating evacuation paths. Various people-counting technologies are proposed in the literature. Passive infrared radiation (PIR) sensors [42] and active infrared sensors [43] are used to detect the presence of occupants in a building. This triggers FPGA boards to notify Raspberry Pi modules, which calculate the direction of movement to update the room’s occupancy count. Image sensors and depth video processing technology is proposed in [44] to detect human heads. The authors of [45] use wireless signals to detect and track human respiration rates when counting people.

For fire evacuations in high-rise buildings, evacuating via stairs instead of elevators is recommended. However, stair congestion is more likely when a crowd is taking the stairs to evacuate [46]. Ref. [47]’s study focuses on partitioning evacuees into groups and calculating their departure times to prevent congestion at exits. To summarize, in existing research, how to design dynamic evacuation signage control schemes to maximize the chance of a successful evacuation and how to generate temperature-safe, smoke-safe, and congestion-safe evacuation routes are still challenges to be addressed. In this paper, we propose an optimization-based dynamic evacuation signage control scheme to address these issues at the same time and then verify it using FDS simulations in the Taipei 101 shopping mall.

## 3. Mathematical Model for Evacuation Sign Control in Multi-Floor and Multi-Exit Buildings

In this section, we propose a mathematical model to capture the evacuation sign control mechanism in multi-floor and multi-exit buildings. The set of nodes and links in the planar graph constructed in the previous section are used as the input parameters for the following mathematical models. The basic idea of this mathematical model is to maximize the number of evacuation signs that indicate the evacuation direction leading to an emergency exit. An evacuation sign can turn on the direction light only when there is a safe evacuation route from the evacuation sign to an emergency exit. If there is no safe evacuation route to any emergency exits, it will not turn on any evacuation direction light. Herein, a safe evacuation route is one that is safe in terms of temperature and smoke along all the links on the evacuation route.

Because fires can spread quickly in fire zones, an evacuation route that is safe at one moment may not be safe at the next moment. Here, we define a time slot as the time interval in which the sensors transmit the sensed temperature and smoke data back to the control center (e.g., thirty seconds). Hence, at every time slot, the control center will send and control the evacuation direction of every evacuation sign based on the sensed data at the last time slot.

In the following, we first propose the Evacuation Sign (ES) mathematical model. Basically, the number of possible evacuation directions of any evacuation sign is based on the layout of the building. For instance, if there are four evacuation directions (right, left, up, and down) as shown in Figure 2, then *I* = {left, right, up, and down}.

### 3.1. Evacuation Sign (ES) Model

First, the notations used in our formulation are as follows:

**Input values:**

G: the set of intelligent evacuation signs;

U: the set of evacuees;

T: the set of time slots during the evacuation;

$Q_{g}$: the set of evacuation paths that are selected by an intelligent evacuation sign g∈G;

$P_{u}$: the set of evacuation paths that are selected by an evacuee u∈U;

L: the set of possible links on the planar graph;

*E*: the set of emergency exit nodes on the planar graph;

I: the set of possible evacuation directions of an intelligent evacuation sign;

*M*: the upper limit of human tolerance in regards to temperature (e.g., 100 °C);

S: the upper limit of human tolerance in regards to smoke;

Ξ: the upper limit of the number of evacuees on a link;

Λ: a very large number;

$\sigma_{lt}$: the temperature at link l∈L at time slot *t* ∈T;

$\varepsilon_{lt}$: the smoke at link l∈L at time slot *t* ∈T;

$\theta_{l}^{i}$: the indication function, where $\theta_{l}^{i}$ = 1 when the direction of link *l* is the *i* element in set *I* and $\theta_{l}^{i}$ = 0 otherwise;

$\alpha_{ql}$: the indication function, where $\alpha_{pl}$ = 1 when the link *l* is on the evacuation path *q* of an intelligent evacuation sign g where $q\in Q_{g}\;and\;g\in G,\;\;\;$ and $\alpha_{pl}$ = 0 otherwise;

$\beta_{qe}$: the indication function, where $\beta_{qe}$ = 1 when the emergency exit *e* is the destination node of the evacuation path *q* selected by the intelligent evacuation sign g where q∈ and g∈G, and = 0 otherwise;

$\rho_{ql}$: the indication function, where $\rho_{ql}$ = 1 when the link *l* is the first link on the evacuation path *q* selected by the intelligent evacuation sign g where $q\in Q_{g}\;and\;g\in G$, and = 0 otherwise;

**Decision variables:**

*w_qt_*: = 1 if an evacuation path $q\in Q_{g}$ is selected by an intelligent evacuation sign g∈G at time slot *t* ∈T and = 0 otherwise;

$j_{glt}$: = 1 if a link l∈L is on the selected evaculation path by an intelligent evacuation sign g∈G at time slot *t* ∈T and = 0 otherwise;

$r_{gt}^{i}$ = 1 if an intelligent evacuation sign g∈G turns on the light with the direction at the *i* element in set *I* at time slot *t* ∈T and = 0 otherwise;

The ES mathematical model formulation is proposed as follows.

Problem (P_1_):

(1) $$Z_{p1}=\max\;\sum_{g\in G}\sum_{t\in T}\sum_{i\in I}r_{gt}^{i}$$

Subject to:

$\sum_{i\in I}r_{gt}^{i}\leq 1$∀g∈G,t∈T(2)$\sum_{q\in Q_{g}}w_{qt}=\sum_{i\in I}r_{gt}^{i}$∀g∈G,t∈T(3)$\sum_{q\in Q_{u}}\sum_{e\in E}w_{qt}\beta_{qe}\leq 1$∀g∈G,t∈T(4)$w_{qt}{\times \alpha }_{ql}\leq j_{glt}$$\forall q\in Q_{g},g\in G,l\in L,t\in T$(5)$j_{glt}{\times \sigma }_{lt}\leq M$∀g∈G,l∈L,t∈T(6)$j_{glt}{\times \varepsilon }_{lt}\leq S$∀g∈G, l∈L,t∈T(7)$\sum_{t\in T}j_{glt}\leq 1$∀g∈G,l∈L(8)${r_{gt}^{i}\leq w}_{qt}{\times \rho }_{ql}\times \theta_{l}^{i}$$\forall q\in Q_{g},g\in G,l\in L,t\in T,i\in I$(9)$w_{qt}$*=* 0 or 1$\forall q\in Q_{g},g\in G,t\in T$(10)$j_{glt}$ = 0 or 1∀g∈G,l∈L,t∈T(11)$r_{gt}^{i}$ = 0 or 1∀g∈G,t∈T,i∈I.(12)

In Problem (P_1_), the objective function is to maximize the total number of evacuation signs by turning on the direction light. Constraint (2) ensures that any evacuation sign g∈G can turn on one direction light at most for every time slot t∈T. Constraint (3) ensures that an evacuation sign turns on the direction light only when an evacuation path exists. In other words, if the evacuation sign g∈G turns on the direction light at the time slot t∈T, then there is an evacuation path for evacuation sign g. In addition, if the evacuation sign g∈G does not turn on the direction light at the time slot t∈T, then there is no evacuation path for evacuation sign g. Constraint (4) ensures that at most one evacuation path with an emergency exit as the destination node is selected by an evacuation sign g∈G at any time slot t∈T. Constraints (2)–(4) jointly ensure that if an evacuation sign selects an evacuation path to an emergency exit, then this evacuation sign must turn on one evacuation direction light. In addition, if the evacuation sign does not turn on the evacuation direction light, then there is no evacuation path to an emergency exit from this evacuation sign.

Constraints (5) and (6) ensure that the temperature on the evacuation path does not exceed the upper limit of human tolerance in regards to temperature *M*. Constraints (5) and (7) ensure that the smoke on the evacuation path does not exceed the upper limit of human tolerance in regards to smoke *S*. Constraints (5)–(7) jointly ensure that the selected evacuation path shown by any evacuation sign is safe in regards to temperature and smoke. Without these two constraints, evacuees might get hurt or even faint on the evacuation path. In other words, these two constraints ensure that evacuees can survive on the evacuation path.

Constraint (8) ensures that any link l∈L is selected by the evacuation path for one time slot at most. Hence, there is no loop in the selected evacuation path for any evacuation sign g∈G. This is to ensure that the evacuees that follow the evacuation sign will not keep circling the fire zone. Constraint (9) ensures that the direction shown on the evacuation sign is consistent with the direction of the evacuation path. Constraints (10)–(12) define the feasible values for the decision variables.

This ES model ensures that if an evacuation sign selects a temperature-safe and smoke-safe evacuation path to an emergency exit, then the evacuation direction light on the evacuation sign that leads to the evacuation path must turn on. By maximizing the number of evacuation signs by turning on the evacuation lights, the ES model maximizes the number of evacuation paths to evacuate as many evacuees as possible. Hence, the ES model is a kind of indirect mathematical model to maximize the number of evacuation paths. Then, a more intuitive mathematical model can maximize the number of evacuation paths in the objective function. In the next section, we show this evacuation path for our evacuation sign mathematical model.

### 3.2. Evacuation Path for Evacuation Sign (EPES) Model

The EPES mathematical model formulation is proposed as follows.

Problem (P_2_):

(13) $$Z_{p2}=\max\;\sum_{t\in T}\sum_{g\in G}\sum_{q\in Q_{g}}w_{qt}$$

Subject to:

$\sum_{i\in I}r_{gt}^{i}\leq 1$∀g∈G,t∈T(14)$\sum_{q\in Q_{g}}w_{qt}=\sum_{i\in I}r_{gt}^{i}$∀g∈G,t∈T(15)$\sum_{q\in Q_{g}}\sum_{e\in E}w_{qt}\beta_{qe}\leq 1$∀g∈G,t∈T(16)$w_{qt}{\times \alpha }_{ql}\leq j_{glt}$$\forall q\in Q_{g},g\in G,l\in L,t\in T$(17)$j_{glt}{\times \sigma }_{lt}\leq M$∀g∈G,l∈L,t∈T(18)$j_{glt}{\times \varepsilon }_{lt}\leq S$∀g∈G, l∈L,t∈T(19)$\sum_{t\in T}j_{glt}\leq 1$∀g∈G,l∈L(20)${r_{gt}^{i}\leq w}_{qt}{\times \rho }_{ql}\times \theta_{l}^{i}$$\forall q\in Q_{g},g\in G,l\in L,t\in T,i\in I$(21)$w_{qt}$*=* 0 or 1$\forall q\in Q_{g},g\in G,t\in T$(22)$j_{glt}$ = 0 or 1∀g∈G,l∈L,t∈T(23)$r_{gt}^{i}$ = 0 or 1∀g∈G,t∈T,i∈I.(24)

The constraints in Problem (P_2_) are all identical to those in Problem (P_1_). The only difference is the objective function. Instead of maximizing the number of evacuation signs by turning on the direction lights, the objective function in Problem (P_2_) is to maximize the number of evacuation paths selected by the evacuation signs to evacuate the evacuees. This objective function is more related to maximizing the number of evacuees that are successfully evacuated compared to Problem (P_1_). However, evacuation congestion is not considered in this EPES mathematical model, so it is not valid for the evacuation of crowds. In the next section, we add the congestion constraint to the EPES mathematical model to derive the Congestion-aware Evacuation Sign Control (CESC) mathematical model.

### 3.3. Congestion-Aware Evacuation Sign Control (CESC) Model

Next, the complete CESC mathematical model is proposed in Problem (P_3_).

Problem (P_3_):

(25) $$Z_{p3}=\max\;\sum_{t\in T}\sum_{g\in G}\sum_{q\in Q_{g}}w_{qt}$$

Subject to:

$\sum_{i\in I}r_{gt}^{i}\leq 1$∀g∈G,t∈T(26)$\sum_{q\in Q_{g}}w_{qt}=\sum_{i\in I}r_{gt}^{i}$∀g∈G,t∈T(27)$\sum_{q\in Q_{g}}\sum_{e\in E}w_{qt}\beta_{qe}\leq 1$∀g∈G,t∈T(28)$w_{qt}{\times \alpha }_{ql}\leq j_{glt}$$\forall q\in Q_{g},g\in G,l\in L,t\in T$(29)$j_{glt}{\times \sigma }_{lt}\leq M$∀g∈G,l∈L,t∈T(30)$j_{glt}{\times \varepsilon }_{lt}\leq S$∀g∈G, l∈L,t∈T(31)$\sum_{t\in T}j_{glt}\leq 1$∀g∈G,l∈L(32)$\sum_{g\in G}j_{glt}\leq \Xi$∀l∈L, t∈T(33)${r_{gt}^{i}\leq w}_{qt}{\times \rho }_{ql}\times \theta_{l}^{i}$$\forall q\in Q_{g},g\in G,l\in L,t\in T,i\in I$(34)$w_{qt}$*=* 0 or 1$\forall q\in Q_{g},g\in G,t\in T$(35)$j_{glt}$ = 0 or 1∀g∈G,l∈L,t∈T(36)$r_{gt}^{i}$ = 0 or 1∀g∈G,t∈T,i∈I.(37)

The link congestion problem happens when a link is selected by too many evacuation signs on their evacuation paths. This can be avoided by restricting a link from being selected by too many evacuation signs on their evacuation paths. In Problem (P_3_), Constraint (33) is added to realize the above idea. Constraint (33) specifies that every link l∈L at a time slot t∈T can be only selected by at most Ξ evacuation signs on their evacuation paths. Note that when there is congestion at the evacuation link, the evacuation time will grow exponentially with respect to the number of evacuees at that link [8]. Hence, by enforcing Constraint (33), we can implicitly prevent longer evacuation times at any link l∈L at any time slot t∈T.

## 4. Solution Approaches

Because of the temperature and smoke constraints in Constraints (29)–(31), Problem (P_3_) needs to identify a constrained shortest path problem for every evacuation sign. This was proven to be an NP-hard problem in [48]. Hence, there is no polynomial time algorithm that can optimally solve Problem (P_3_). We propose a heuristic algorithm, called Evacuation Sign Planning (ESP), to tackle Problem (P_3_). The basic idea of the ESP algorithm is to identify the shortest temperature-safe, smoke-safe, and congestion-aware evacuation path for every evacuation sign and then guide all the evacuees via the direction lights on the evacuation signs to evacuate. The link arc weight is set as the combination of the hop count and danger index. Note that Λ is a very large number. Then, the danger index $\Omega_{lt}$ at link l∈L at time t∈T is defined as the following equation.

(38) $$\Omega_{lt}=1+\sigma_{lt}/M+\varepsilon_{lt}/S+\left(\sum_{g\in G}j_{glt}\right)/\Xi \;\;\;\sigma_{lt}<M,\;\varepsilon_{lt}<S\;\mathrm{and}\;\sum_{g\in G}j_{glt}\geq \Xi$$

(39) $$\Omega_{lt}=\Lambda \;\;\;\sigma_{lt}\geq M\;or\;\varepsilon_{lt}\geq S.$$

In Equation (38), there are four components in the danger index $\Omega_{lt}$ at link l∈L at time t∈T. The first component (i.e., 1) is the hop count, which implies that a shorter hop count to an emergency exit is preferred over a longer hop count when identifying an evacuation path. The second component is the temperature danger index. In [2], the upper limit of human tolerance in regards to temperature is 100 °C. We set *M* = 100, and $\sigma_{lt}$ is the temperature in Celsius at link l∈L at time t∈T. The third component is the smoke danger index. In [37], the smoke upper limit of human tolerance in regards to smoke is 0.5 FED. Thus, we set *S* = 0.5, and $\varepsilon_{lt}$ is the FED index value at link l∈L at time t∈T.

The fourth component in Equation (38) is the congestion danger index, $\left(\sum_{g\in G}j_{glt}\right)/\Xi$. The term $\left(\sum_{g\in G}j_{glt}\right)$ calculates how many evacuation lights select link l∈L on their evacuation path at time t∈T. Hence, larger $\left(\sum_{g\in G}j_{glt}\right)$ values indicate that more evacuation lights have selected link l∈L on their evacuation path and more evacuees will be guided to link l at time t. As more evacuees are gathered at link l, the congestion probability at link l increases. By using the shortest path algorithm to identify the evacuation path for each evacuation sign, the link with a bigger value of $\left(\sum_{g\in G}j_{glt}\right)/\Xi$ will be unlikely to be selected on the evacuation path. Then the evacuation congestion problem can be circumvented.

The link arc weight settings in Equations (38) and (39) consider the interplay of the distance to the emergency exit, temperature danger index, smoke danger index, and congestion danger index. At the early stage of a fire, when the temperature danger index and smoke danger index are very small, the shortest route to an emergency exit is selected as the evacuation path. Note that by using the shortest path to select the evacuation path for each evacuation sign, it is very likely that some links (e.g., staircases) will be selected on the evacuation paths of many evacuation signs resulting in congestion. If more evacuees are guided to the congested link, it might lead to a stampede that incurs injuries and even deaths in the evacuation. By introducing the fourth term in Equation (38), the congested links will not be selected at the next time slot to prevent a stampede. The temperature danger index and the smoke danger index come into play at the latter stage of the fire’s progression so that temperature and smoke safety are key to identifying the evacuation path. Hence, in the early stage of the fire, it identifies the shortest evacuation path to an emergency exit that will evacuate as many evacuees as possible. Then, in the latter stage of the fire, the identified evacuation path takes into account the dangers of high temperature and smoke. To summarize, by introducing the hop count, temperature danger index, smoke danger index, and congestion danger index in the arc weight, Dijkstra’s algorithm identifies the fastest temperature-safe, smoke-safe, and congestion-aware path to an emergency exit.

We first propose the ESP algorithm (Algorithm 1) to solve Problem (P_3_). The basic goal of the ESP algorithm is to identify a temperature-safe, smoke-safe, and congestion-aware evacuation path for each evacuation sign, then turn on the evacuation light that leads in the direction of the selected evacuation path.
**Algorithm 1: ESP****Begin****Initialize** the value of all the decision variables to be zero;**Let** the node position of an evacuation sign be the source node;**Let** *t* = 0;**While** $\left(\left(t\leq \left|T\right|\right)\;\right)$//looping at each time slot *t***Begin** **Get** the sensed temperature data and smoke data on each link *l* at time slot t; **Calculate** link arc weight $\Omega_{lt}$ defined in Equations (38) and (39) for each link *l* at time slot *t*; **For** every evacuation sign g∈G
 **Begin**  **Let**
$j_{glt}$= 0 ∀l∈L; //initialization, no selected links on the evacuation path at time slot *t*  //Stage 1: identify the shortest path for evacuation sign g to each emergency exit  **Let**
$\mu_{1}$ = 0;  **Let**
$\mu_{2}$ = Λ;  **For** every exit e∈E;  **Begin**   **Perform** Dijkstra’s shortest path cost from evacuation sign g to emergency exit e;   **Let**
$\lambda_{ge}$ be the shortest path cost from evacuation sign g to emergency exit e by using the Dijkstra’s algorithm;   **If** ($\lambda_{ge}<\mu_{2}$)//shortest path to emergency exit *e* has lower cost   **Begin**    $\mu_{2}=\lambda_{ge}$;    $\mu_{1}=e$;//emergency exit $\mu_{1}$ has lower cost   **End**//If  **End**//For every exit  **//Stage 2:** turn on g’s direction light to exit $\mu_{1}$
  **If** ($\mu_{1}$ == 0)//cannot find any temperature-safe and smoke-safe path to all the exits  **Begin**   **Let**
$r_{gt}^{i}$ = 0 ∀i∈I; //does not turn on the direction light  **End**//If ($\mu_{1}$ == 0)  **Else**//there is a temperature-safe and smoke-safe path to exit $\mu_{1}$, turn on the direction light to exit $\mu_{1}$
  **Begin**   **Let**
$r_{gt}^{i}$ = 0 ∀i∈I; //initialization   **Let**
$r_{gt}^{i}$ = 1 where direction light *i* leads to the evacuation path to emergency exit $\mu_{1}$; //turn on the direction light that leads to the evacuation path to emergency exit $\mu_{1}$
   **Set**
$j_{glt}$ =1 for every link l∈L on the selected evacuation path to exit $\mu_{1}$
  **End**//Else **End**//For every evacuation sign **Let**
t=t+1; //proceed to next time slot**End**;//While loop**End**

There are two stages in the ESP algorithm. The first stage in the ESP algorithm identifies the shortest path for each evacuation sign. Since there are multiple emergency exits for each evacuation sign, first the shortest path to each emergency exit is computed via Dijsktra’s shortest path algorithm, and the emergency exit with the minimum cost is identified as the destination. In the second stage, the direction light on the evacuation sign to the emergency exit with the minimum cost and shortest path is turned on. In the ESP algorithm, at each time slot, the direction light on the evacuation signs will be turned on to guide the evacuees to the selected evacuation paths.

The time complexity for the ESP algorithm is determined by the “While” loop for each time slot. And the time complexity for the “While” loop is determined by the Stage 1 because of the shortest path algorithm. For each evacuation sign, Dijkstra’s shortest path algorithm needs be performed with each emergency exit as a destination node and then the emergency exit with the minimum path cost can be identified. The time complexity of Dijkstra’s algorithm is $O\left({\left|G\right|}^{2}\right),$ where $\left|G\right|$ is the number of evacuation signs in the networks. Then, the time complexity for stage 1 in the “While” loop is $O\left(\left|E\right|\times {\left|G\right|}^{2}\right)$. Because of the “For” loop, all three stages must loop for $\left|G\right|$ times inside the “While” loop. Then, the total time complexity for the ESP and MESP algorithms is $O\left(\left|T\right|\times \left|E\right|\times {\left|G\right|}^{3}\right),$ where $\left|T\right|$ is the number of time slots.

## 5. Computational Experiments and Simulation Results

To verify the quality of the results of the proposed ESP algorithm, we compare it with two existing algorithms. The first one is the Fixed Evacuation Light (FEL) algorithm, which is adopted in existing fixed evacuation systems. In buildings with multiple emergency exits, for each evacuation sign, first the shortest path to each emergency exit is computed via Dijsktra’s shortest path algorithm and the emergency exit with the minimum cost is identified as the destination. Then, the direction of the evacuation sign is set to the nearest emergency exit. Hence, the basic objective of the FEL is to guide evacuees to the nearest emergency exit. Note that in the FEL algorithm, evacuation signs are fixed regardless of the fire spreading in the building, which is widely adopted in existing evacuation system.

The second algorithm is the Random Selection (RS) algorithm, in which the evacuees randomly choose a link for evacuation. Basically, the RS algorithm is applicable to buildings in which there is no guidance from evacuation signs. In this case, the evacuees can only randomly select an evacuation route to evacuate. It can be expected that the RS algorithm might lead to a longer evacuation route due to evacuees walking randomly around the building. Therefore, in the upcoming experiments, we will assess the effectiveness of the proposed ESP algorithm by comparing it with existing evacuation scenarios. The initial scenario involves fixed evacuation signs, wherein the evacuation direction to the nearest emergency exit is determined using the FEL algorithm. The second scenario assumes the absence of evacuation signs, leading evacuees to randomly choose an evacuation direction (referred to as the RS algorithm). Through the subsequent experiments, we demonstrate that the ESP algorithm consistently outperforms both the FEL algorithm (representing existing fixed evacuation signs) and the RS algorithm (representing scenarios without evacuation signs) in terms of the probability of successful evacuation across all test cases.

**Figure 3:**
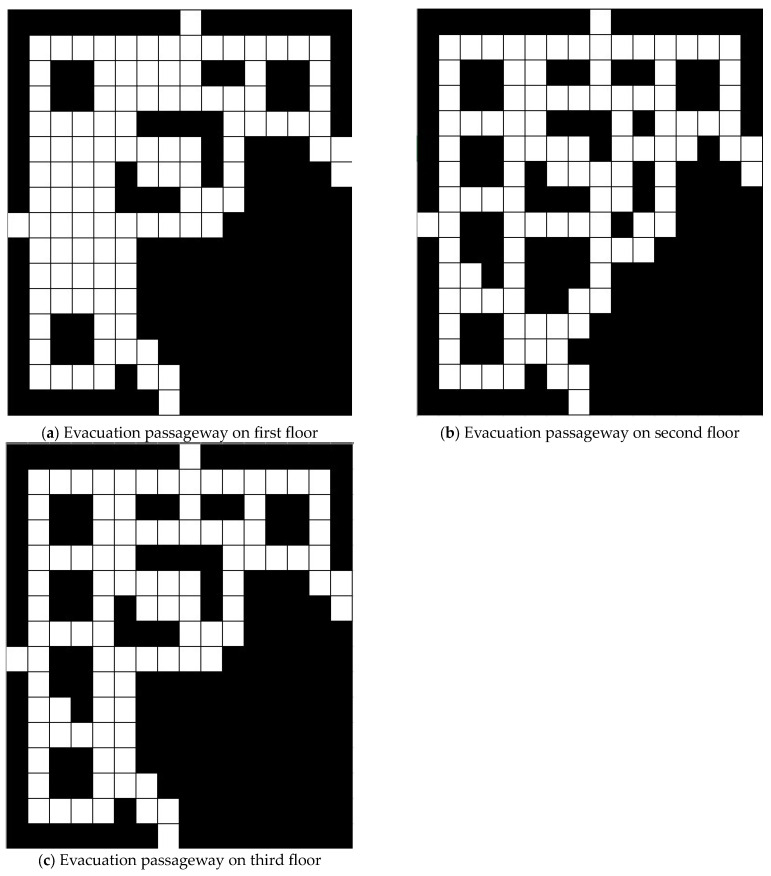
Evacuation passageway map at Taipei 101 shopping mall.

To model the spread of fire and smoke in the fire zone, we utilize the widely recognized Fire Dynamics Simulator (FDS 7.9.1), a software developed by the National Institute of Standards and Technology (NIST) under the United States Department of Commerce [33]. We select the Taipei 101 mall as the location for our fire simulation due to its distinction as the tallest building in Taiwan and the fact it is visited by millions of people annually. Figure 3 shows the evacuation passageway map for each floor of the Taipei 101 shopping mall, where there are four emergency exits on the first floor. In Figure 3, the white block is the passageway. The bottom-right area in Figure 3 is an office that is not connected to the shopping mall.

In the FDS, the size at each floor of the Taipei 101 mall is 160 m times 160 m, and the shopping mall area from the first floor to the third floor make up three-fourths of the total area. The number of deployed sensors with smoke- and temperature-sensing capabilities is 294, and the spacing between the sensors is 10 m. In the shopping mall, 48 sprinklers are installed on each floor with 20 m of spacing in between them. Two fire ignitors are simulated in the FDS: methane and polyvinyl chloride (PVC). Methane ignition usually comes from stove fires in kitchens. PVC ignition usually comes from electric short circuiting. We used the same configurations for the two fire ignitors in the FDS as shown in [8]. In the FDS, the simulation time is 68 iterations, in which each iteration is 27 s. The total simulation time is 1836 s.

We use Equation (40) to calculate the evacuation time on a link with respect to the number of evacuees. First, we set the link capacity in the Taipei 101 mall to be 25. This implies that if the number of evacuees on a link is not greater than 25, then all the evacuees can evacuate to the next node. However, if the number of evacuees on a link exceeds 25, then it is congested, and Equation (40) is used to calculate the number of iterations needed for all the evacuees to evacuate to the next node.

(40) Φλ=1,                                                          if λ≤251+Φλ−λ2λ−2525,          if λ>25

**Figure 4:**
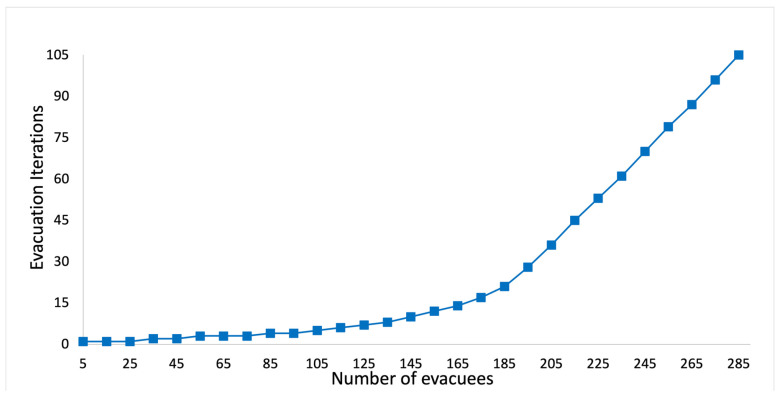
Evacuation iterations with respect to the number of evacuees.

Equation (40) is a recursive function where $\Phi \left(\lambda \right)$ calculates the evacuation iterations when there are λ evacuees on a link. When λ>25, 2λ−2525 calculates the original number of iterations needed to evacuate λ number of evacuees; λ2λ−2525 calculates the number of evacuees that can be evacuated in that iteration; and λ−λ2λ−2525 calculates the remaining number of evacuees at that link. For example, if λ=105, then originally it needs 2105−2525= 10 iterations for the evacuees to evacuate to the next node. In that iteration, 1052105−2525=10 evacuees would be evacuated to the next node, and there would be 95 evacuees remaining at that link. The next iteration calculates $\Phi \left(95\right)$. Finally, five iterations are needed to evacuate all 105 evacuees to the next node. Figure 4 shows the evacuation iterations with respect to the number of evacuees using Equation (40).

In the first set of computational experiments, the evacuees are uniformly distributed into the locations of 294 sensor nodes. In [8], we show that the temperature danger zone is a subset of the smoke danger zone when a fire occurs. In other words, risk of smoke presides over the risk of heat during the fire evacuation. In the following experiments, we will consider the smoke danger threshold as an FED index score = 0.3 for children and the elderly and as a FED index score = 0.5 for healthy adults [49].

**Figure 5:**
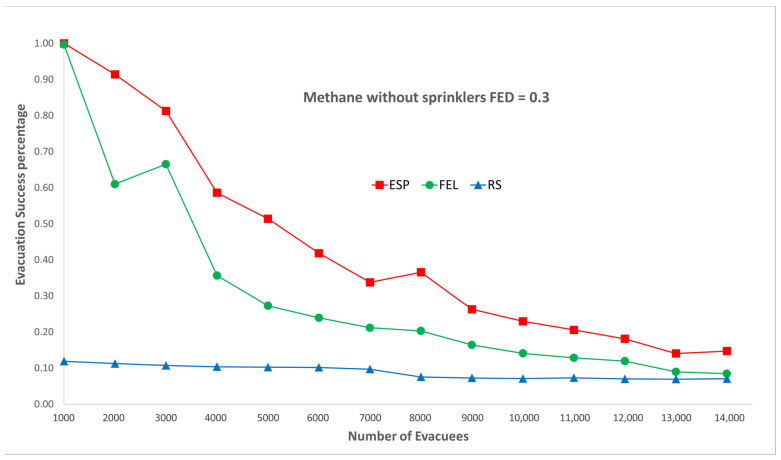
Methane fire without sprinklers, FED = 0.3.

**Figure 6:**
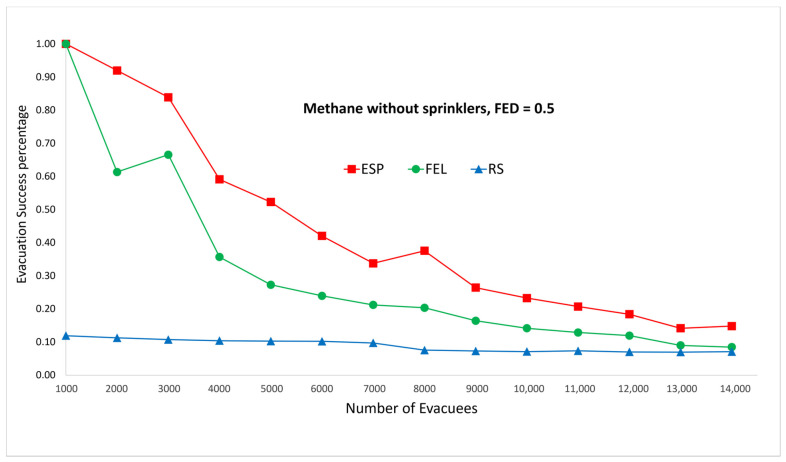
Methane fire without sprinklers, FED = 0.5.

Figure 5 and Figure 6 show performance comparisons in the context of a methane fire when the sprinklers are not activated. The ESP algorithm is superior to the FEL and the RS algorithms in terms of the probability of evacuees successfully evacuating, in particular when there are 2000 evacuees in the shopping mall. For 2000 evacuees, the evacuation success probabilities are 91%, 61%, and 11% for the ESP, FEL, and RS algorithms, respectively. The second interesting result is that probability of evacuees successfully evacuating is almost the same for a FED index score = 0.3 and FED = 0.5. This indicates that the smoke danger index in Equation (38) has a very small contribution to the danger index $\Omega_{lt}$ at link l∈L at time t∈T in a methane fire without sprinklers.

**Figure 7:**
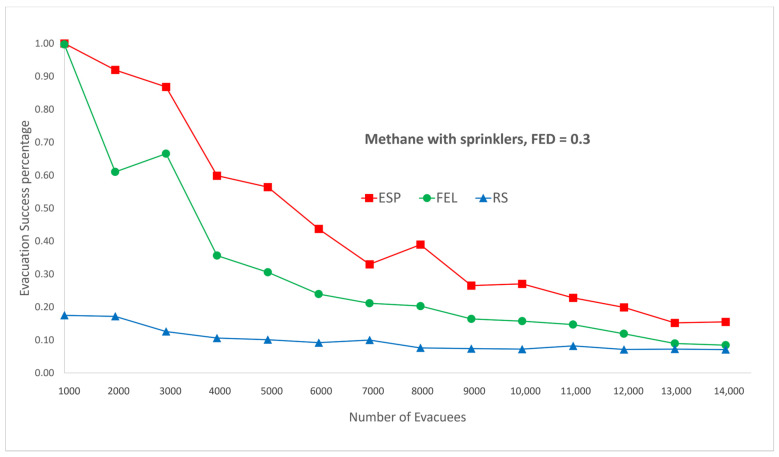
Methane fire with sprinklers, FED = 0.3.

**Figure 8:**
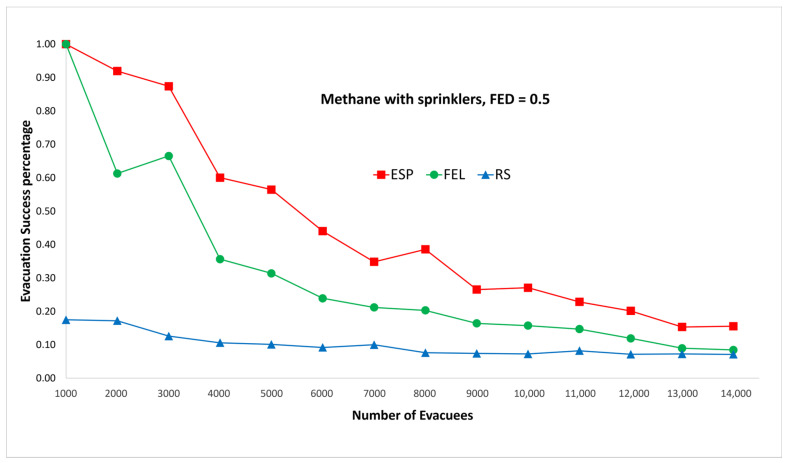
Methane fire with sprinklers, FED = 0.5.

Figure 7 and Figure 8 show the performance comparisons in the context of a methane fire when the sprinklers are activated. Again, the ESP algorithm is superior to the FEL and RS algorithms in terms of the probability of evacuees successfully evacuating, in particular when there are 2000 evacuees in the shopping mall. For 2000 evacuees, the probabilities of successfully evacuating are 92%, 61%, and 17% for the ESP, FEL, and RS algorithms, respectively. The probability of evacuees successfully evacuating in Figure 7 and Figure 8 is almost the same. This indicates the smoke danger index in Equation (38) has a very small contribution to the danger index $\Omega_{lt}$ at link l∈L at time t∈T whether the sprinklers are activated or not. As compared to Figure 5 and Figure 6, there is only a marginal improvement of the probability of evacuees successfully evacuating using the ESP and FEL algorithms when activating the sprinklers. However, there are substantial improvements when using the RS algorithm and activating the sprinklers. Another interesting result is that the ESP and FEL algorithms both have a 100% evacuation success rate with/without sprinklers when there are 1000 evacuees. This indicates that 1000 occupants is the safest capacity in the context of a methane fire at the Taipei 101 shopping mall.

**Figure 9:**
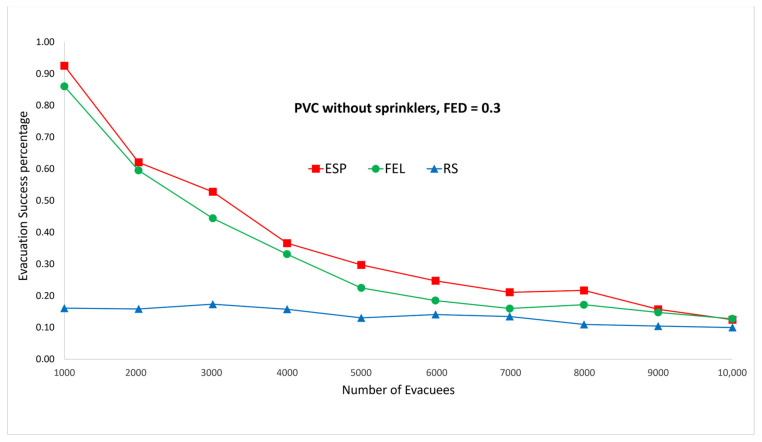
PVC fire without sprinklers, FED = 0.3.

**Figure 10:**
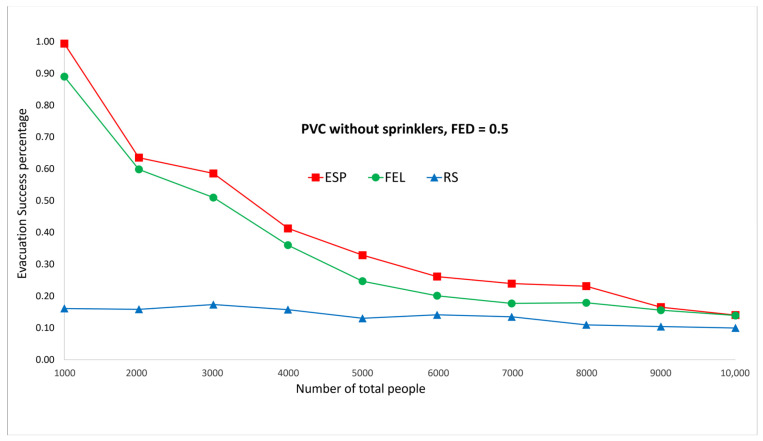
PVC fire without sprinklers, FED = 0.5.

Figure 9 and Figure 10 show the performance comparisons in the context of a PVC fire when the sprinklers are not activated. The ESP algorithm is superior to the FEL and RS algorithms in terms of the probability of evacuees successfully evacuating, in particular when there are 1000 evacuees in the shopping mall. When there are 1000 evacuees, the probabilities of successfully evacuating with an FED index score = 0.5 are 100%, 89%, and 16% for the ESP, FEL, and RS algorithms, respectively. It can be observed that the probability of evacuees successfully evacuating with an FED index score = 0.5 is better than that with an FED index score = 0.3 for the ESP algorithm. In particular, when there are 1000 evacuees, the probability of evacuees successfully evacuating with an FED index score = 0.3 is 93% as compared to 100% with an FED index score = 0.5 for the ESP algorithm.

**Figure 11:**
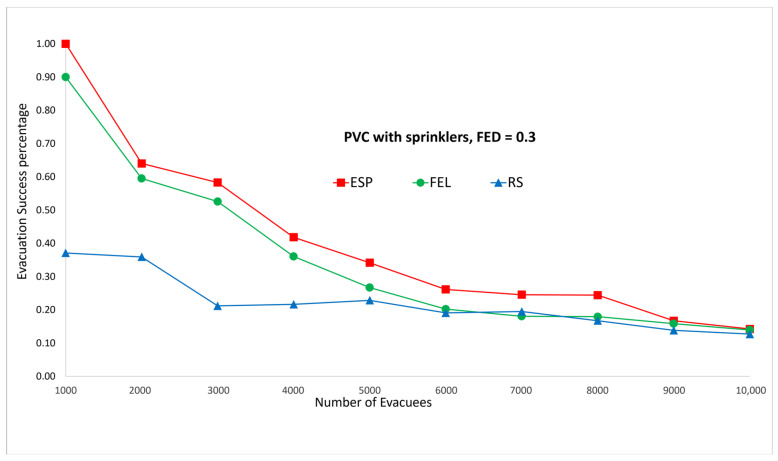
PVC fire with sprinklers, FED = 0.3.

**Figure 12:**
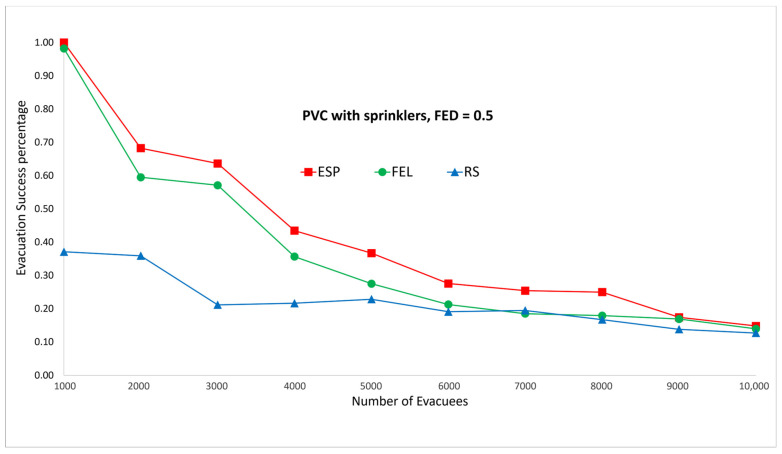
PVC fire with sprinklers, FED = 0.5.

Figure 11 and Figure 12 show the performance comparisons in the context of a PVC fire when the sprinklers are activated. The ESP algorithm is superior to the FEL and RS algorithms in terms of the probability of evacuees successfully evacuating, in particular when there are 1000 evacuees in the shopping mall with an FED index score = 0.3. When there are 1000 evacuees, the probabilities of evacuees successfully evacuating with an FED index score = 0.3 are 100%, 90%, and 37% for the ESP, FEL, and RS algorithms, respectively. The probability of evacuees successfully evacuating for the ESP algorithm at an FED index score = 0.5 is better than that at an FED index score = 0.3. When there are 3000 evacuees, the probabilities of evacuIfully evacuating are 58% and 64% at FED index scores = 0.3 and = 0.5, respectively. This indicates that the smoke danger index is more important in the context of PVC fires both with/without sprinklers. Another interesting point is that the probability of evacuees successfully evacuating in the context of a PVC fire is lower than that in methane fire for the ESP algorithm. This is because PVC fires are more toxic than methane fires, so smoke from PVC fires will reach the FED threshold faster than smoke from methane fires.

**Figure 13:**
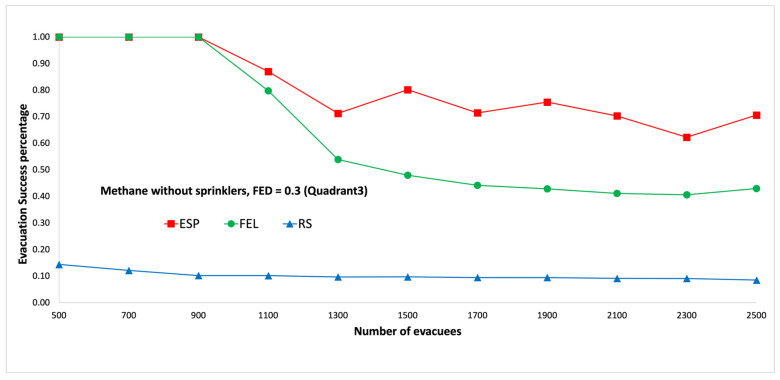
Methane fire without sprinklers, FED = 0.3 (the third quadrant).

**Figure 14:**
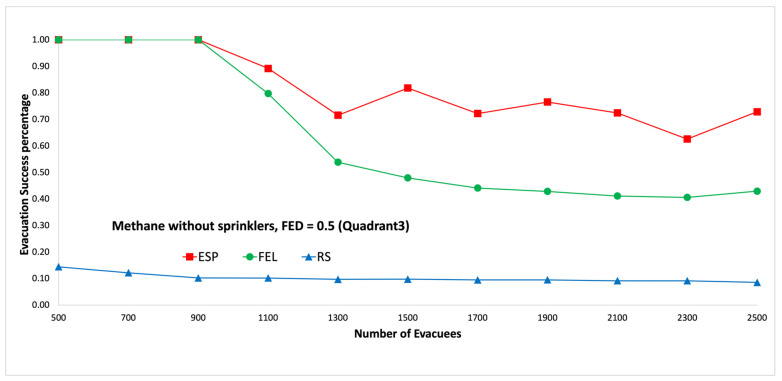
Methane fire without sprinklers, FED = 0.5 (the third quadrant).

In the second set of experiments, the evacuees are uniformly distributed to the third quadrant, which is at the bottom-left area on each floor. Note that the fire ignitor is in the third quadrant on the first floor. Then, it can be expected that there will be more congestion at the two emergency exits, which are located in the third quadrant on the first floor. Figure 13 and Figure 14 show performance comparisons in the context of a methane fire when the sprinklers are not activated. The ESP algorithm is superior to the FEL and RS algorithms in terms of the probability of evacuees successfully evacuating, in particular when there are 1500 evacuees in the shopping mall. When there are 1500 evacuees, the probabilities of evacuees successfully evacuating at an FED index score = 0.5 are 82%, 48%, and 10% for the ESP, FEL, and RS algorithms, respectively. The probability of evacuees successfully evacuating for an FED index score = 0.5 is 2% better than that at an FED index score = 0.3 for the ESP algorithm when the number of evacuees is not smaller than 1100.

**Figure 15:**
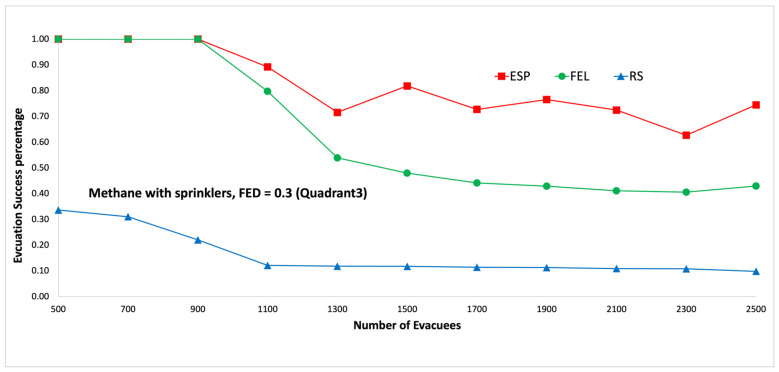
Methane fire with sprinklers, FED = 0.3 (the third quadrant).

**Figure 16:**
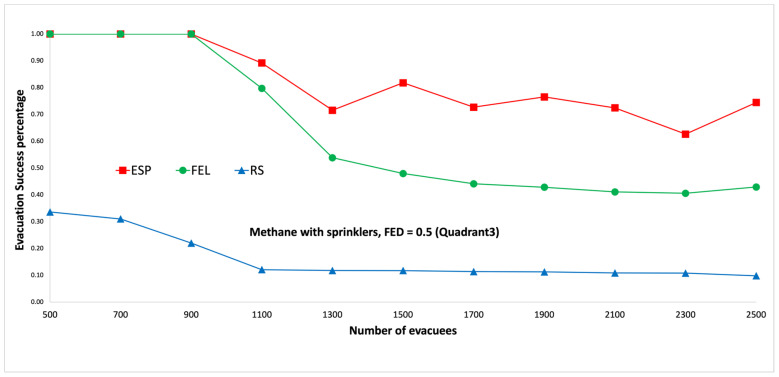
Methane fire with sprinklers, FED = 0.5 (the third quadrant).

Figure 15 and Figure 16 show performance comparisons in the context of a methane fire when the sprinklers are activated. Again, the ESP algorithm is superior to the FEL and RS algorithms in terms of the probability of evacuees successfully evacuating, in particular when there are 1500 evacuees in the shopping mall. When there are 1500 evacuees, the probabilities of evacuees successfully evacuating are 82%, 48%, and 12% for the ESP, FEL, and RS algorithms, respectively. The probabilities of evacuees successfully evacuating in Figure 15 and Figure 16 are almost the same. This indicates the smoke danger index in Equation (38) has a very small contribution to the danger index $\Omega_{lt}$ at link l∈L at time t∈T, whether the sprinklers are activated or not. Another interesting result is that the ESP and FEL algorithms both have a 100% evacuation success rate with/without sprinklers when the number of evacuees is not greater than 900 evacuees.

**Figure 17:**
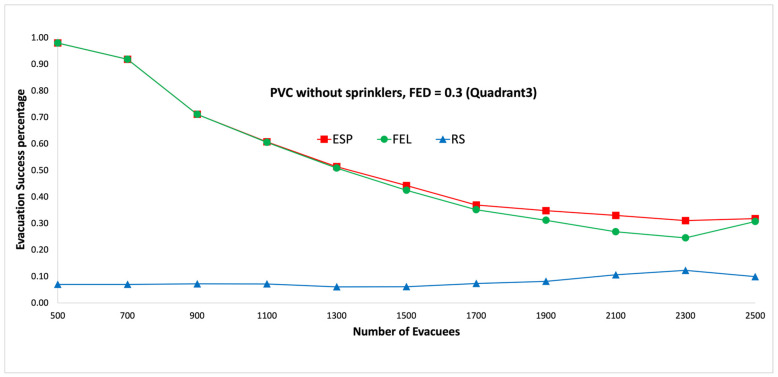
PVC fire without sprinklers, FED = 0.3 (the third quadrant).

**Figure 18:**
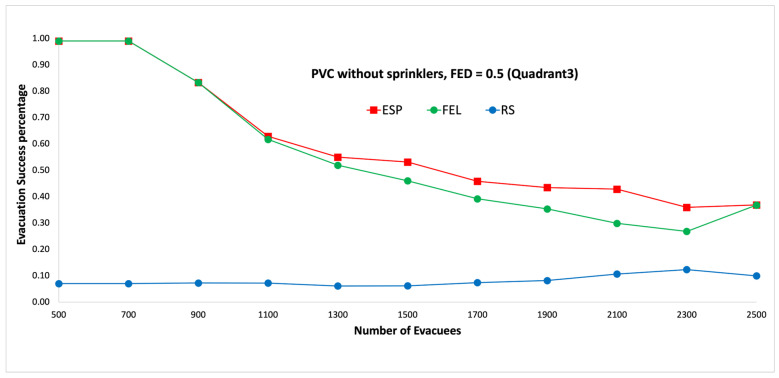
PVC fire without sprinklers, FED = 0.5 (the third quadrant).

Figure 17 and Figure 18 show the performance comparisons in the context of a PVC fire when the sprinklers are not activated. The ESP algorithm is superior to the FEL and RS algorithms in terms of the probability of evacuees successfully evacuating, in particular when there are 1000 evacuees in the shopping mall. When there are 2100 evacuees, the probabilities of evacuees successfully evacuating at an FED index score = 0.5 are 43%, 30%, and 11% for the ESP, FEL, and RS algorithms, respectively. It can be observed that the probability of evacuees successfully evacuating at an FED index score = 0.5 is better than that at an FED index score = 0.3 for the ESP algorithm. In particular, when there are 900 evacuees, the probability of evacuees successfully evacuating at an FED index score = 0.3 is 71% compared to 83% at an FED index score = 0.5 for the ESP algorithm.

**Figure 19:**
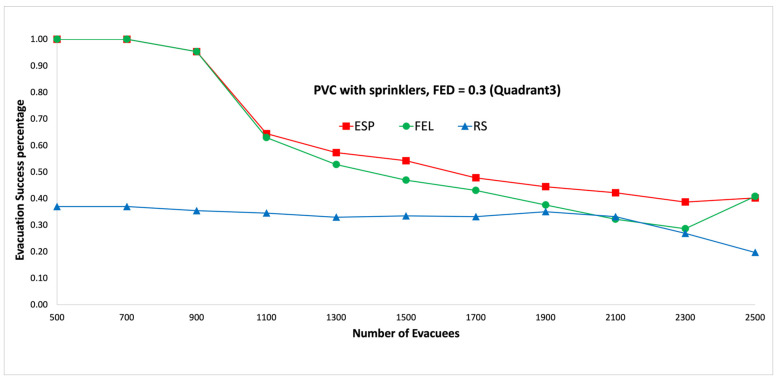
PVC fire with sprinklers, FED = 0.3 (the third quadrant).

**Figure 20:**
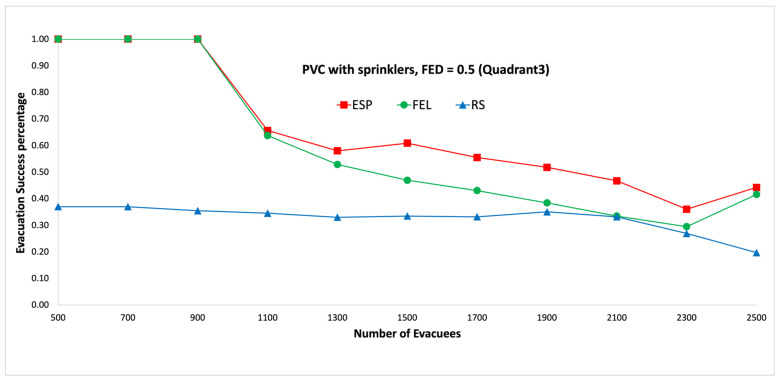
PVC fire with sprinklers, FED = 0.5 (the third quadrant).

Figure 19 and Figure 20 show performance comparisons in the context of a PVC fire when the sprinklers are activated. The ESP algorithm is superior to the FEL and RS algorithms in terms of the probability of evacuees successfully evacuating. When there are 1500 evacuees, the probabilities of evacuees successfully evacuating at an FED index score = 0.5 are 61%, 47%, and 33% for the ESP, FEL, and RS algorithms, respectively. The probability of evacuees successfully evacuating for the ESP algorithm at an FED index score = 0.5 is better than that at an FED index score = 0.3. When there are 1900 evacuees, the probabilities of evacuees successfully evacuating are 44% and 52% at FED index scores = 0.3 and = 0.5, respectively. This indicates that the smoke danger index is more important in the context of PVC fires both with/without sprinklers. The probability of evacuees successfully evacuating in PVC fires is lower than that in methane fires for the ESP algorithm. This is because PVC fires are more toxic than methane fires, so smoke from PVC fires will reach the FED threshold faster than smoke from methane fires.

Comparing the results in the first set and second set of experiments, we can make the following observation. The biggest gap between the ESP and FEL algorithms in the first set of experiments is for the methane fire with sprinklers (Figure 7 and Figure 8), in which the gap is 31% (92% vs. 61%) when there are 2000 evacuees. The average gap is 12%. And the biggest gap between the ESP and FEL algorithms in the second set of experiments is for the methane fire with sprinklers (Figure 15 and Figure 16), in which the biggest gap is 34% (82% vs. 48%) when there are 1500 evacuees. The average gap is 19%. Hence, the ESP algorithm can perform much better than the FEL algorithm when there is more congestion during an evacuation from a methane fire.

The biggest gap between the ESP and FEL algoritms in the first set of experiments is for the PVC fire with sprinklers (Figure 11 and Figure 12), in which the gap is 9% (37% vs. 28%) when there are 5000 evacuees. The average gap is 5.6%. And the biggest gap between the ESP and FEL algorithms in the second set of experiments is for the methane fire with sprinklers (Figure 19 and Figure 20), in which the biggest gap is 14% (52% vs. 38%) when there are 1900 evacuees. The average gap is 6%. Hence, the ESP algorithm can perform much better than the FEL algorithm when there is more congestion during an evacuation from a PVC fire.

To ensure a timely response, the computational duration plays a crucial role in the ability of the proposed algorithm to promptly relay evacuation directions for dynamically controlled evacuation signs. Specifically, IoT-enabled temperature and smoke sensors consistently transmit their sensed data to the IIFESS server in Figure 2, either through wired or wireless signals, at each iteration. Subsequently, the proposed algorithms within the IIFESS server compute real-time evacuation directions to manage each evacuation sign within a single time slot. It is noteworthy that in the FDS simulation platform, each iteration spans 27 s.

**Figure 21:**
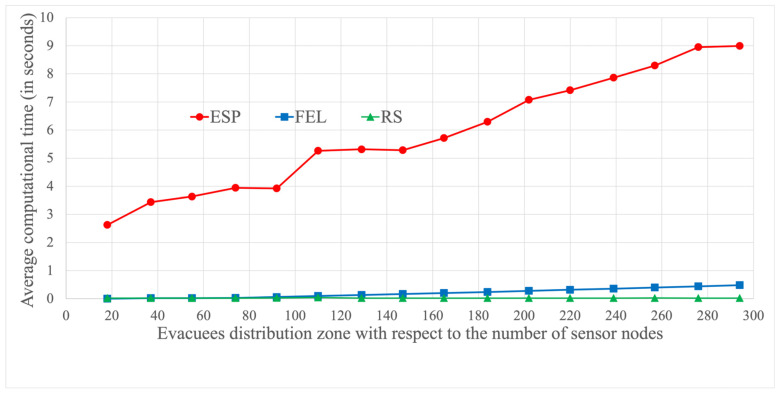
Comparisons of average computational time for one iteration.

To elaborate, the IoT sensors gather temperature and smoke data at 27 s intervals, and the computational time must stay within this timeframe to regulate the evacuation signs based on the most recent sensed data. Figure 21 illustrates each proposed algorithm’s average computational time required for one iteration, all within 27 s intervals. The hardware specifications for executing these algorithms, as depicted in Figure 21, include a CPU with a 2.5 GHz Intel i5-13500, 16 GB DDR4 RAM, and a GPU with RTX4070-12G.

In Figure 21, we compare the average computational time for each proposed algorithm for each iteration (i.e., 27 s), considering the distribution of evacuees within the Taipei 101 shopping mall. There are a total of 294 sensors, with the area covered by these sensors divided into 16 deployment zones, each covered by 19 sensor nodes (calculated as 294/16). For example, the evacuees are initially uniformly distributed across the deployment zone in the first set of experiments, covered by 294 sensor nodes. In the second set of experiments, the evacuees are placed in the third quadrant of the deployment zone, which is covered by 74 sensor nodes (calculated as 294/4).

It is important to note that the computational time is consistently zero in the RS algorithm, in which the evacuees randomly choose a link to evacuate without an evacuation sign. In contrast, for the ESP and FEL algorithms, the average computational time is a monotonically increasing function concerning the number of sensor nodes. As the evacuees are initially deployed in a larger zone, more evacuation signs are needed to indicate the evacuation direction, resulting in an increased computational time. The longest computational time occurs when the evacuees are uniformly distributed throughout the Taipei 101 mall (covered by 294 sensor nodes). In this scenario, the ESP algorithm requires 9 s of computational time, while the FEL algorithm needs 0.5 s—both well within the 27 s iteration time. In summary, the three proposed algorithms can effectively guide evacuees based on the most recent sensed temperature and smoke data.

## 6. Conclusions

In today’s evacuation systems, the direction of evacuation signs are fixed and always guide evacuees to the nearest emergency exit. However, these systems are not adaptable to rapidly changing fire situations if the nearest emergency exit is on fire. With the help of smoke sensors and temperature sensors, more intelligent evacuation signs can be realized to guide evacuees to emergency exits quickly and safely. In this paper, we propose an intelligent evacuation sign control mechanism to set the direction light on evacuation signs to dynamically adapt to the fire’s spread. We built this evacuation sign control mechanism using a rigorous mathematical formulation and devised an ESP heuristic to identify the shortest temperature-safe, smoke-safe, and congestion-aware evacuation path for every evacuation sign and then set the direction light to that path. Fire simulations based on FDS software were conducted using the Taipei 101 shopping mall to obtain temperature and smoke data at each sensor node with respect to a methane fire and a PVC fire. Based on data from the sensors, the proposed ESP algorithm outperforms the existing FEL algorithm and the RS algorithm both when evacuees are uniformly distributed throughout the shopping mall and when evacuees are only distributed in the third quadrant of the shopping mall. The RS algorithm in which evacuees evacuate randomly has the worst performance. When the evacuees are only distributed in the third quadrant of the shopping mall, congestion is the main reason preventing evacuees from evacuating successfully. The congestion danger index in the ESP algorithm sets the direction light on each evacuation sign to guide the evacuees away from sites with congestion. The ESP algorithm outperforms the FEL algorithm in terms of the probability of evacuees successfully evacuating by up to 34% in the context of a methane fire and 14% in the context of a PVC fire. In addition, the probability of evacuees successfully evacuating using the ESP algorithm is more significant in evacuations with more congestion compared to the FEL algorithm.

## Data Availability

All the data of this study are available and included within the manuscript itself.
